# Vaccine-induced tumor regression requires a dynamic cooperation between T cells and myeloid cells at the tumor site

**DOI:** 10.18632/oncotarget.4940

**Published:** 2015-08-11

**Authors:** Maxime Thoreau, HweiXian Leong Penny, KarWai Tan, Fabienne Regnier, Julia Miriam Weiss, Bernett Lee, Ludger Johannes, Estelle Dransart, Agnès Le Bon, Jean-Pierre Abastado, Eric Tartour, Alain Trautmann, Nadège Bercovici

**Affiliations:** ^1^ Inserm, U1016, Institut Cochin, Paris, France; ^2^ Cnrs, UMR8104, Paris, France; ^3^ Université Paris Descartes, Sorbonne Paris Cité, Paris, France; ^4^ Equipe labellisée “Ligue contre le Cancer”, Paris, France; ^5^ Singapore Immunology Network, BMSI, A-STAR, Singapore; ^6^ Institut Curie, Paris, France; ^7^ INSERM U1143, Paris, France; ^8^ CNRS UMR3666, Paris, France; ^9^ Inserm U970, PARCC, Université Paris Descartes, Sorbonne Paris Cité, Paris, France

**Keywords:** T lymphocytes, myeloid cells, vaccine, tumor regression, imaging

## Abstract

Most cancer immunotherapies under present investigation are based on the belief that cytotoxic T cells are the most important anti-tumoral immune cells, whereas intra-tumoral macrophages would rather play a pro-tumoral role. We have challenged this antagonistic point of view and searched for collaborative contributions by tumor-infiltrating T cells and macrophages, reminiscent of those observed in anti-infectious responses. We demonstrate that, in a model of therapeutic vaccination, cooperation between myeloid cells and T cells is indeed required for tumor rejection. Vaccination elicited an early rise of CD11b^+^ myeloid cells that preceded and conditioned the intra-tumoral accumulation of CD8^+^ T cells. Conversely, CD8^+^ T cells and IFNγ production activated myeloid cells were required for tumor regression. A 4-fold reduction of CD8^+^ T cell infiltrate in CXCR3KO mice did not prevent tumor regression, whereas a reduction of tumor-infiltrating myeloid cells significantly interfered with vaccine efficiency. We show that macrophages from regressing tumors can kill tumor cells in two ways: phagocytosis and TNFα release. Altogether, our data suggest new strategies to improve the efficiency of cancer immunotherapies, by promoting intra-tumoral cooperation between macrophages and T cells.

## INTRODUCTION

T lymphocytes play an important role in restraining tumor development. Their infiltration in most human primary tumors is associated with better prognosis [1] and they seem to keep in check disseminated tumor cells and metastatic outgrowth [2]. Based on these notions, various types of T-cell based immunotherapies have been developed for treating cancer patients, and provided encouraging clinical results in the past few years. In particular, melanoma patients have been successfully treated with high doses of IL-2 following adoptive transfer of TIL (Tumor infiltrating T lymphocytes) and lympho-depletion by chemo- and radiotherapy [3]. Leukemic patients have been treated with autologous T cells engineered to express anti-CD19 chimeric receptors [4]. Other impressive results have been achieved in patients with advanced melanoma treated with a combination of anti-PD-1 and anti-CTLA-4 antibodies [5].

At the same time where these successes were obtained, the dominant point of view attributed a major role for T cells in the anti-tumor defense and most often restricted the contribution of macrophages to their suppressive and pro-tumoral potential. The temporal dimension of anti-tumoral immune responses is too rarely taken into account, which leads to neglect a major fact: cell states are dynamic. The immune system has been shaped by evolution so as to fight infections, not cancer, and efficient immune responses against viruses or bacteria invariably require a whole set of cellular interactions. When these pathogens are cleared, the immune responses are transient, with an acute and a recovery phase that are quite different, and both necessary for the efficacy and the safety of the system [6].

It is conceivable that a similar biphasic time-response would apply for an immune reaction leading to tumor rejection. More than a century ago, W. Coley was one of the first to show that responses to infections could be followed by tumor regression, without clearly understanding why [7]. Adjuvants like CpG have been used to mimic such an inflammation in the context of tumors, but have often been considered mostly as an efficient way of maturing antigen-presenting cells to facilitate T cell priming. Such a view centered on T cells has overlooked the notion that innate cells like macrophages and neutrophils can also be efficient killers, for instance during the acute phase of anti-infectious responses.

A fundamental question that remains to be solved is how immune cells can reject tumor cells embedded in a tumor mass, in a connective network of stromal cells. In particular, the role of T lymphocytes in the rejection of such nodules is far from being understood. A classical view poses that T cells kill tumor cells directly in the tumor. Dynamic imaging revealed indeed that TCR-transgenic T cells can be found close to dying tumor cells after adoptive transfer [8] and apoptosis induced by T cells in contact with tumor cells could be monitored directly *in vivo* [9]. It is difficult however to estimate how important these events are during tumor regression. One must take into account that this process is relatively slow, since one T cell needs several hours to kill one tumor cell [9]. This may explain why adoptive transfer of large numbers of T cells or chimeric receptor-transfected T cells is necessary to induce objective clinical responses in solid tumors (i.e., partial or complete tumor regression). Without adoptive transfer of such massive quantities of T cells, TIL are largely outnumbered by tumor cells, and it is highly unlikely that they would exhibit a massive direct cytotoxic effect. One must therefore consider more likely that T cells interact and cooperate with other immune cells that could gain cytotoxic potential against tumor cells to reject an established tumor.

It is striking that the ability of infiltrating T cells to secrete IFNγ appeared more crucial than their perforin-dependent cytotoxicity in various cancer models [10, 11]. This observation suggested that other cytotoxic effector cells may indeed be activated due to IFNγ-producing T cells. Our group has previously shown that in advanced human tumors, T cells accumulate in the peri-tumoral stroma, and are rarely in direct contact with tumor cells [12]. It is thus likely that T cells mostly interact with other immune cells in the stroma. Intriguingly, frequent contacts between T cells and myeloid cells in tumors have been reported [13]. The functional consequences of such interactions remain unclear although they are generally considered to be non productive in progressing tumors [14, 15].

Previous studies have focused on progressing tumors and mechanisms of immune failure. By contrast, the goal of this work was to study the dynamics of an efficient anti-tumoral immune response occurring in regressing tumors. Drawn from observations of immune responses during infections, we co-administered IFNα with a vaccine, in the TC1 tumor transplantation model. The vaccine was composed of a delivery system targeting dendritic cells, the non-toxic B-subunit of Shiga toxin coupled to HPV16 derived-E7 peptide (STxBE7 or E7-vaccine), and was used to elicit CD8^+^ T cells specific for E7 antigen expressed by the TC1-tumor cell line [16]. Vaccination of these tumor-bearing mice induced tumor regression, and by monitoring the influx of immune cells into tumors preceding regression, we have identified the key cellular and molecular players mediating the anti-tumor immunity. Using different experimental approaches, we provide evidence that, at least in this model and in the EG7 model, not only T cells but also activated, cytotoxic, tumor infiltrating myeloid cells are required for eliminating the tumor by TNFα production and phagocytosis of tumor cells. In these models, the key factor for the anti-tumoral action is not one cell type, but a dynamic and multi-step *cooperation* between two cell types.

## RESULTS

### The combination of E7-vaccine + IFNα induces systematic regression of TC1-tumors

**Figure 1:**
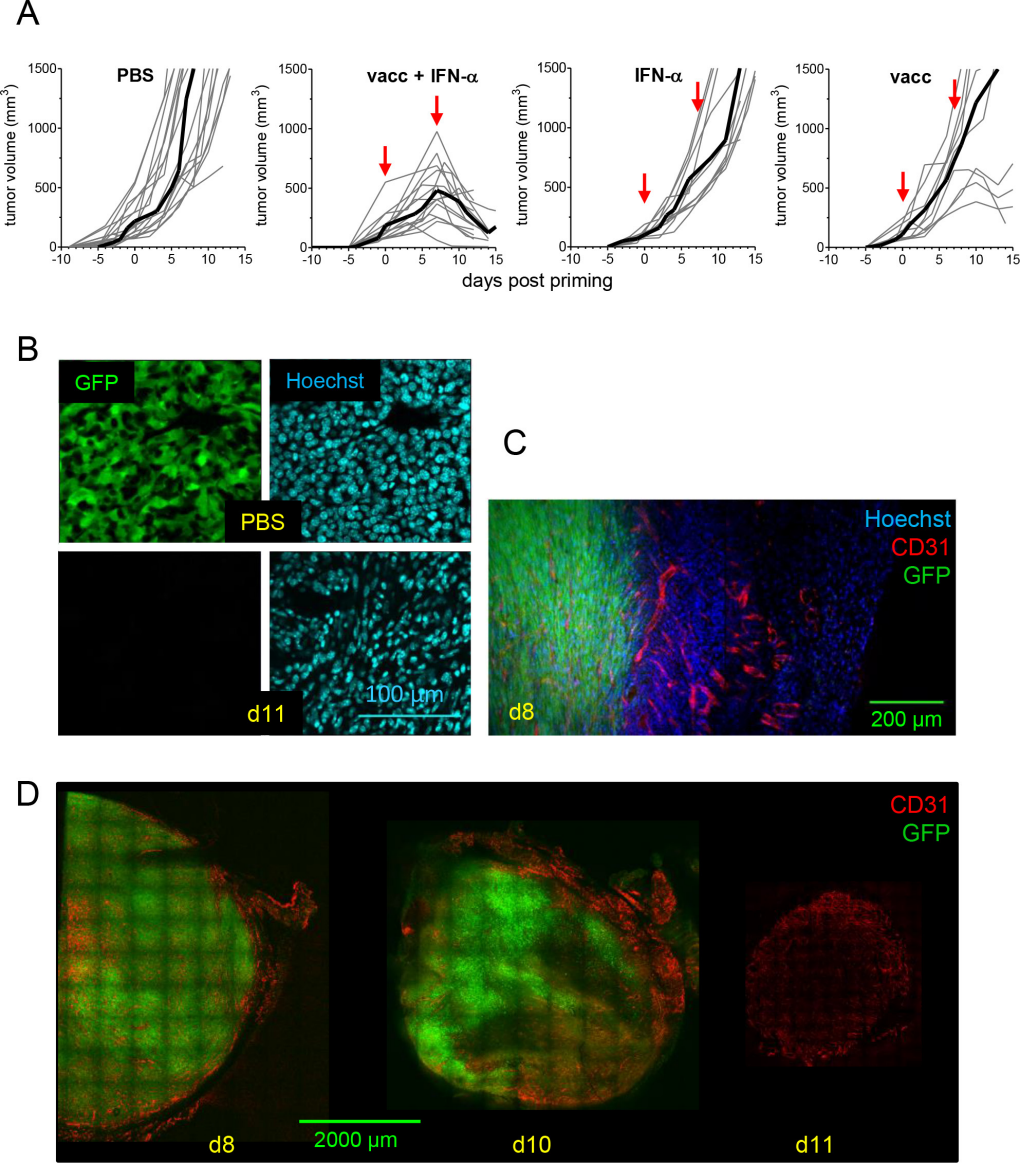
TC1 tumor regression is triggered by injection of E7-vaccine + IFNα C57BL6/J mice with TC1-tumors received various combinations of the E7-vaccine and IFNα. **A.** Tumor growth curves (individual mice, grey thin line; mean, dark thick line) are shown for each treatment. Red arrows : days of priming (day 0) and boost (day 7). **B.** The disappearance of tumor cells after vaccination is viewed 11 days after the priming as the disappearance of GFP^+^ cells (left) and as the decrease in the size of the nuclei (right), tumor nuclei being clearly larger (top) than nuclei of infiltrating immune cells (bottom). **C.** The disappearance of GFP^+^ cells (d8) begins in the tumor peripheral regions, **D.** which show a dense CD31^+^ vasculature (d8) and then affects more internal regions (d10), still the most vascularized ones. At d11, all GFP^+^ cells have disappeared.

C57BL/6J mice were transplanted with TC1 tumor cells expressing the E7 protein from HPV. When tumor nodules reached 6 mm in diameter (∼10 days), mice were treated with two peri-tumoral injections of STxBE7- (termed E7-vaccine thereafter) and IFNα, one week apart. All mice showed a regression of TC1 tumors after the second injection (Figure 1A). Injection of IFNα alone did not halt the tumor growth and in mice treated with the vaccine alone tumors either stabilized or progressed, but almost never regressed after the boost (Figure 1A). These data show that the delivery of the vaccine together with IFNα (mimicking an infection near the tumor site) was optimal for inducing a systematic regression of tumors.

To follow the fate of TC1 tumor-cells *in situ* during tumor regression, mice were transplanted with TC1-GFP cells and tumors were analyzed by immunofluorescence on tumor slices [17]. In contrast to PBS-control mice, most tumor cells had been already eliminated in vaccinated mice at day 11, and replaced by cells with small nuclei, likely immune cells (Figure 1B). Imaging of whole tumor slices revealed that during stabilization at day 8 post-vaccination, tumor attack occurred from the periphery of the tumor (GFP^neg^ area) where the vasculature was denser (Figure 1C and 1D). At day 10, the tumor was also attacked from the inside and finally by day 11, only a dense vascular network, no longer surrounded by GFP^+^ cells, was detected. These results confirmed that tumor vessels, which are required for tumor growth, are also the entry door for the Trojan horse, i.e. anti-tumor immune cells.

### Influx of myeloid cells coincides with the onset of tumor regression and precedes infiltration by CD8^+^ T cells

**Figure 2:**
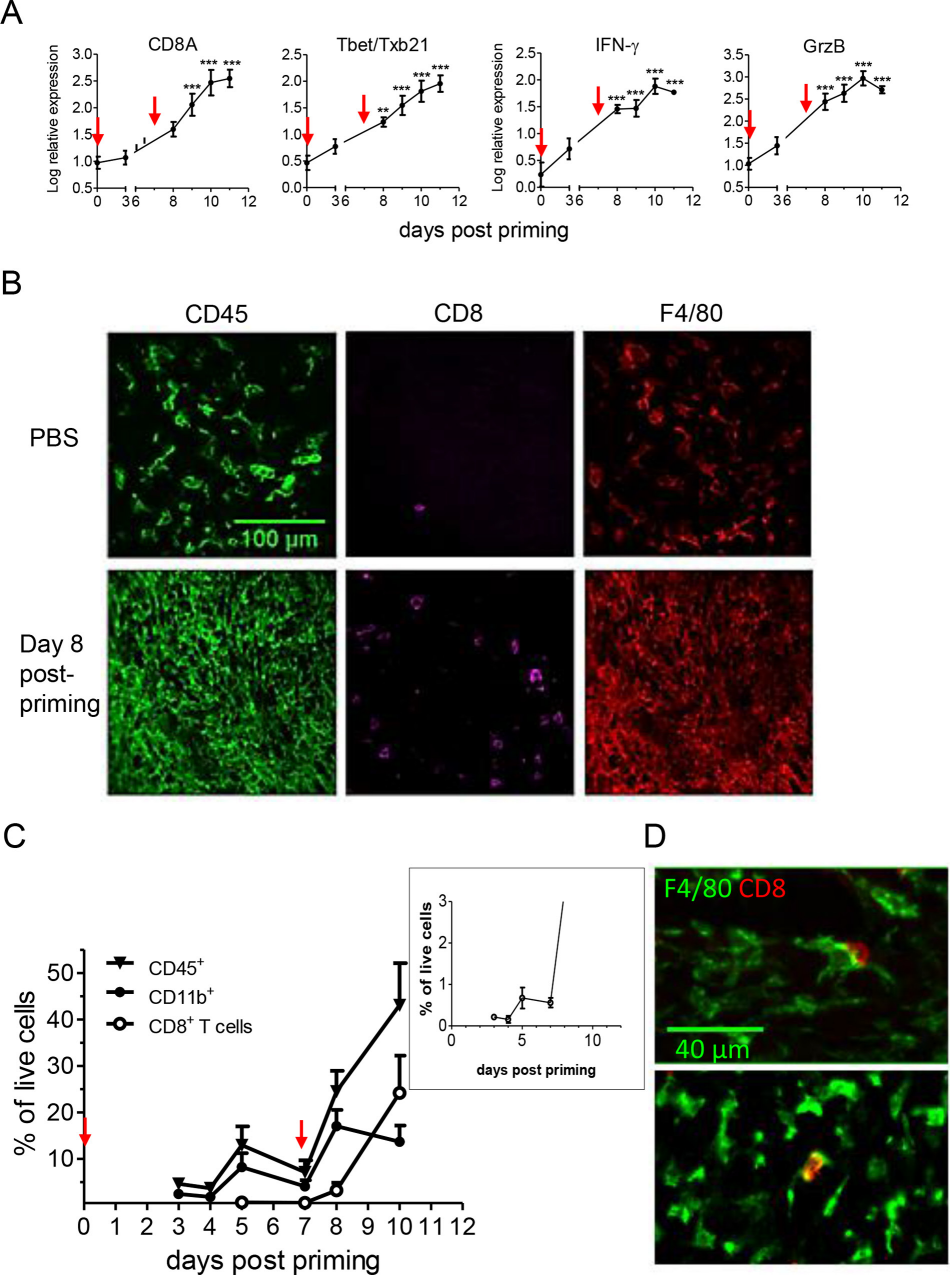
After vaccination with E7-vaccine + IFNα, an increase in the CD11b^+^ myeloid infiltrate precedes the accumulation of CD8^+^ T cells **A.** Transcriptomic analysis of tumor infiltrate collected at various time points after vaccination show a progressive infiltration by type-1 effector CD8^+^ T cells. Time 0 corresponds to aged-matched PBS treated mice in which gene expression was quite stable during the follow up period. Statistical differences between individual time points and time 0 are shown. **B.** In control mice (top, PBS), the CD45^+^ infiltrate is mainly constituted of myeloid F4/80^+^ cells. After vaccination (bottom), regressing tumors (day 8) show a high density of CD45^+^ cells with both CD8^+^ T cells and F4/80^+^ cells. **C.** A detailed kinetics analysis by flow cytometry of CD45^+^, CD11b^+^ and CD8^+^ T cell infiltration after vaccination revealed an early rise in CD11b^+^ cells at day 5. A tiny increase of CD8 T cells, from 0.2 to 0.7% of live cells in the tumor, occurred in this time frame (Inset). **D.** Two examples showing that these rare CD8^+^ T cells at day 5 are in frequent interaction with F4/80^+^ cells.

As T cells were the primary target of the E7-vaccine, we first focused on the T cell response induced by vaccination. Transcriptomic analysis showed that expression of *CD8A* together with *TBX21*, *IFNG* and granzyme B progressively increased between day 8 and day 11 in vaccinated animals (Figure 2A). These data indicate that the compound vaccine primed type 1 cytotoxic CD8^+^ T cells. *In situ*, CD8^+^ T cells started to infiltrate the tumors by day 8 (Figure 2B). Surprisingly however, these CD8 T cells represented only a small portion of the CD45^+^ immune infiltrate detected at that time. While myeloid F4/80^+^ cells were already present in the tumor before treatment, we observed that they formed a much denser network around tumor cells in vaccinated mice compared to T cells (Figure 2B).

As tumor regression in mice treated with E7-vaccine + IFNα occurred in a predictable and reproducible manner (Figure 1A), it was possible to analyze the composition of the immune infiltrate *before* the onset of tumor regression. As shown in Figure 2C, immune cells were recruited to tumors of vaccinated mice in two distinct waves. A first rise in the CD45^+^ cells infiltrate occurred 5 days after priming, increasing from 5% to 12% (a significant increase, *p* = 0.03, Mann-Whitney) and a second rise at day 11, when CD45^+^ cells represented up to 40% of viable cells in the tumor. The kinetics of CD11b^+^ cells followed the same pattern, with an early rise in CD11b^+^ cells 5 days after priming. At this time point, CD11b^+^ cells represented 8% of living cells, and constituted the vast majority of CD45^+^ cells in the tumor, whereas infiltrating CD8^+^ T cells were scarce and represented less than 1% of living cells. At the time when the tumor started to shrink (day 8–9), CD8^+^ T cells were still poorly represented (<5% of live cells) and were largely outnumbered by CD11b^+^ cells (which reached 15% of living cells). Nevertheless, there was a very small increase in CD8^+^ T cells at day 5 (from 0.2 to 0.7% of living cells, right box Figure 2C) and these very rare CD8 T cells made frequent contact with myeloid cells as illustrated in Figure 2D. The density of CD8^+^ T cells became important only at late time points (day 10) when most of the tumor mass had already regressed. Such levels point out an indirect role of these cells, but not a role as major direct cytotoxic effector cells. Other immune cell subsets (CD4^+^ T cells, NK cells, Ly6G^+^ cells and γδT cells) remained at low levels (< 4% of living cells, Figure S1) at all time points. Altogether, these data show that tumor regression is associated with the presence of an early dense myeloid infiltrate in the tumor followed by a secondary accumulation of CD8^+^ T cells.

### Both CD8^+^ TIL and infiltrating-F4/80^+^ cells are necessary for the tumor regression

**Figure 3:**
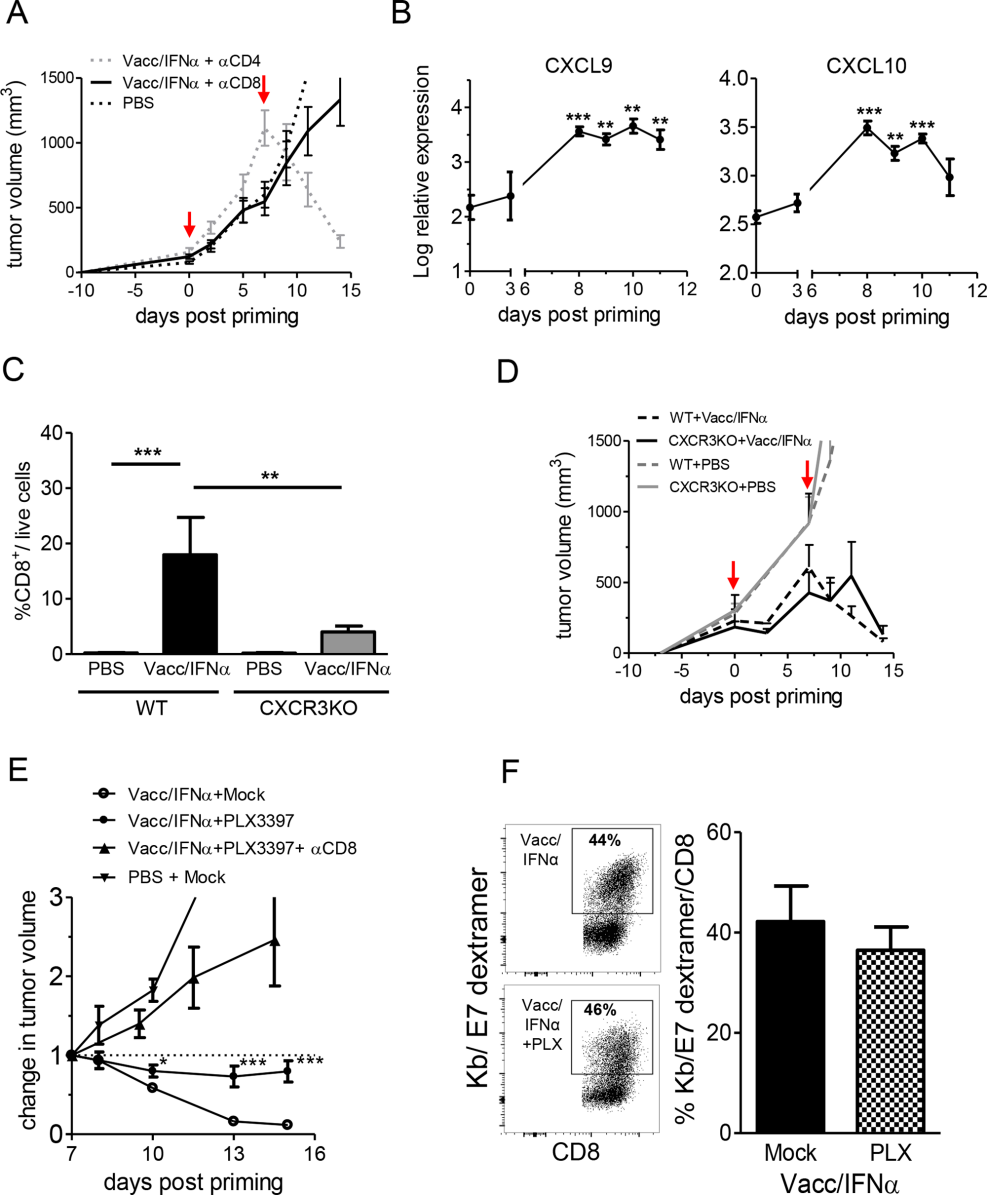
CD8^+^ T cells and myeloid-cell infiltrate are necessary for the tumor regression **A.** Depletion of CD8 T cells prevented vaccine induced-tumor regression whereas depletion of CD4^+^ T cells did not. Tumor growth curves (mean of 8–12 individual mice) are shown for anti-CD8 Ab, anti-CD4 Ab and PBS-control littermates. **B.** Transcriptomic analysis shows that CXCL9 and CXCL10 are upregulated in tumors after E7-vaccine+IFNα treatment. **C.** Reduction in vaccine-induced CD8^+^ T cell infiltrate in CXCR3KO mice compared to wild type mice. **D.** The growth of untreated TC1 tumors is unaffected by the KO of CXCR3. The vaccine treatment induced tumor regression in CXCR3KO just like in wild type littermates. **E.** After depletion of myeloid cells with PLX3397, the E7-vaccine+IFNα only elicited tumor stabilization, but not the tumor regression observed in mice fed with the control chow. Tumor regression was further blocked in mice treated with PLX3397 and anti-CD8 antibody. The change in tumor volume at various days post priming compared to tumor size at day 7 is shown (mean+/−SEM from 5 to 8 mice from 2–3 independent experiments). **F.** Depletion of myeloid cells with PLX3397 did not prevent the priming of Kb/E7-specific CD8 T cells by E7-vaccine, as quantified (left, one typical experiment) with dextramers on CD8 TIL from mock (top) or PLX3397-treated mice (bottom). Right : quantification done in 2 independent experiments.

CD8^+^ TIL were rare when the tumor started to shrink at day 8–9, despite a clear activation and infiltration later on. These data made us wonder whether CD8^+^ TIL were really essential for tumor regression. To address this question, CD8^+^ T cells were depleted at the time of regression, i.e. starting anti-CD8 i.p. injections on day 5 after priming of TC1 tumor bearing mice. All tumors progressed in CD8-depleted animals (Figure 3A). In comparison, depletion of CD4 T cells did not inhibit tumor regression (Figure 3A). These results showed that the presence of CD8, but not that of CD4 T cells, was necessary for TC1 tumor regression.

Our transcriptomic analysis revealed that T-cell chemoattractants CXCL9 and CXCL10 were strongly upregulated after vaccination (Figure 3B). For this reason, we treated chemokine receptor 3 (CXCR3) knockout mice with the compound vaccine. Surprisingly, despite the marked 4-fold reduction in CD8^+^ T cell infiltrate (Figure 3C), TC1-tumors were quite efficiently rejected in vaccinated CXCR3KO mice (Figure 3D). Altogether, these results show that CD8^+^ T cells were important for TC1 tumor regression, but they cannot be the sole cytotoxic effectors at the tumor site.

In order to investigate whether myeloid cells participate in the elimination of tumor cells *in vivo*, we treated TC1-tumor bearing mice with the compound vaccine and PLX3397, a CSF1-R signaling inhibitor [18]. This treatment led to an average 2-fold reduction of intra-tumoral CD11b^+^ cells within 2–3 days, with a significant reduction (∼5-fold) in the proportion of F4/80^+^ cells (Figure S2). In these conditions, the tumors were stabilized, i.e., tumor regression no longer took place (Figure 3E). The simultaneous depletion of CD8^+^ T cells and F4/80^+^ cells abolished the efficacy of the vaccine. Of note, the combination of PLX3397 + anti-CD8 was not more efficient than anti-CD8 alone, because the anti-CD8 alone has already reached a full anti-vaccine efficiency. The frequencies of E7-specific CD8^+^ T cells infiltrating tumors were similarly high in macrophage-depleted and control mice (∼45% of CD8^+^ TIL on day 10, Figure 3F), which demonstrated that this treatment did not interfere with priming by the vaccine compound. It does not mean that the absolute number of CD8 T cells was not affected within the tumor, and in fact, it was decreased. This number results from the combination of the priming efficacy (which was kept intact) and of the efficiency in the recruitment of activated T cells in the tumor (whether E7-specific or not), which was diminished, as a direct or indirect result of the depletion of intra-tumoral macrophages. Altogether, these results show that activated tumor-infiltrating myeloid cells are necessary for TC1-tumor cell elimination after vaccination, and that their action depends on the presence of anti-tumor CD8^+^ T cells.

To strengthen this conclusion, we used another way of reducing the intra-tumoral myeloid infiltrate, by implanting the tumors in CCR2-deficient mice, a condition well known to lower intra-tumoral monocyte recruitment [19]. Under these conditions, the intra-tumoral myeloid infiltrate was reduced, and so was the efficacy of the anti-tumoral vaccine (Figure S3A), which confirmed the necessity of myeloid cells for tumor regression. We investigated further whether anti-tumor myeloid cells could also be induced after immunization in additional tumor models. This turned out to be the case for EG7-tumors (EL4 thymoma cells expressing the chicken ovalbumin antigen) in which regression induced by STxB-OVA+IFNα is dependent on tumor-infiltrating myeloid cells (Figure S3B).

### Dynamic cooperation between intra-tumoral CD8^+^ T cells and myeloid cells

**Figure 4:**
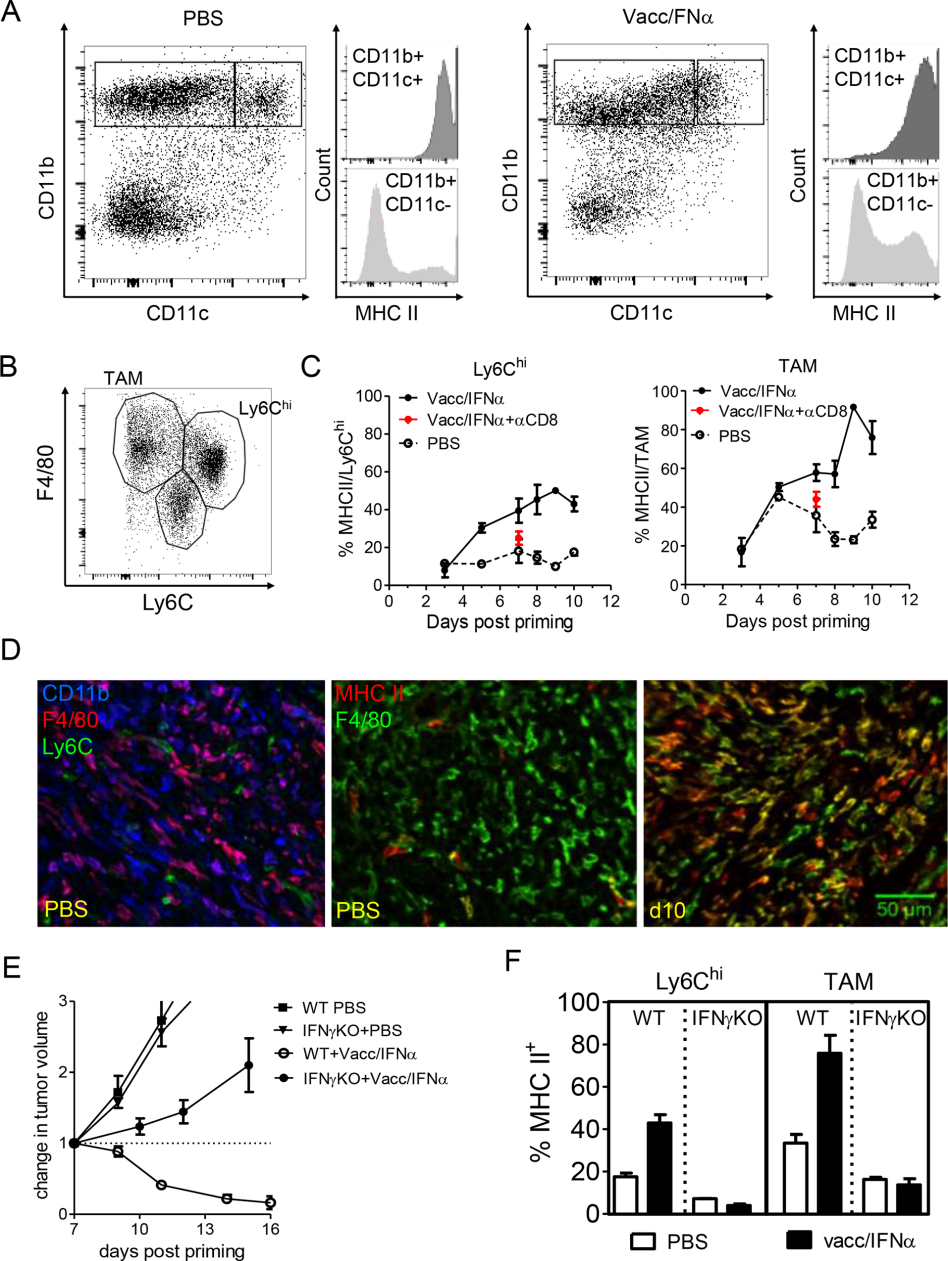
Local activation of F4/80^+^ myeloid cells is promoted by the interactions with IFN-γ producing CD8 T cells **A.** In progressing tumors (PBS, left), a fraction of CD11b^+^ cells also express CD11c and MHC II, but most CD11b^+^ cells do not express MHC II. After vaccination (right, d11 in this example), an increased fraction of CD11b cells express MHC II. **B.** Among CD11b^+^ myeloid cells, at least 3 populations may be distinguished based on F4/80 and Ly6C expression. **C.** After vaccination, MHC II expression is increased both in Ly6C^hi^ cells and in F4/80^hi^ Ly6C^neg^ TAM, except in mice depleted in CD8 (red asterix). **D.** Left : in progressing tumors (PBS), a large fraction of CD11b^+^ cells express F4/80 (different hues of purple). Middle: in progressing tumors, very few macrophages (F4/80^+^) are activated (MHC II^+^). MHC II^+^ F4/80^neg^ cells are even rarer. Right : at day10, a majority of F4/80^+^ cells are MHC II^+^. **E.** The efficacy of E7-vaccine + IFNα treatment is abrogated in IFNγ KO mice (tumor growth curves with the mean+/−SEM of 7 mice from 2 independent experiments). **F.** Activation of Ly6C^hi^ cells and TAM was prevented at day 10 after priming in IFNγ KO mice (mean+/−SEM of 3 mice from 3 independent experiments).

We then analyzed in more details the phenotype and activation status of myeloid cells infiltrating regressing tumors. The CD11b^+^-infiltrate was mainly composed of CD11b^+^CD11c^neg/lo^ and to a lesser extent of CD11b^+^CD11c^hi^ cells (∼10%) (Figure 4A, dot plots). Expression of MHC II molecule was upregulated on CD11b^+^ cells after treatment (Figure 4A, histograms) indicating that these cells were activated. The CD11b^+^CD11c^neg/lo^ infiltrate was composed of F4/80^hi^ Ly6C^lo^ cells (tumor-associated macrophages, TAM) and Ly6C^hi^ F4/80^lo^ cells (Ly6C^hi^, inflammatory monocytes) subsets (Figure 4B). In the absence of vaccination, a minority of Ly6C^hi^ cells (∼10%) expressed MHC II (Figure 4C). The level of MHC II on TAM was more variable along the same period in control mice (from 20–40% MHC II^+^) suggesting that TAM could be locally activated by the growing tumor. However, vaccination induced a strong upregulation of MHC II expression at day 5 on Ly6C^hi^ cells, concomitant with the early rise in myeloid cell number depicted in Figure 2C. Myeloid activation progressively increased to reach a maximum at day7 (∼45% of Ly6C^hi^ cells were MHC II^+^). MHC II expression on TAM was upregulated a bit later (∼day 8) and was expressed by most of these cells (>80%) by day 10. As illustrated in Figure 4D, a large fraction of CD11b^+^ cells expressed F4/80 in progressing tumors (PBS), whereas CD11b^+^ Ly6C^+^ cells were scarce. The major vaccination-induced increase in MHC II expression by F4/80^+^ cells is also shown. It is striking that activation of myeloid cells at day 7 did not occur in animals depleted of CD8^+^ T cells (Figure 4C), indicating that, despite their paucity at that time, vaccine primed-CD8^+^ T cells, that were frequently in close contact with F4/80^+^ cells at this early time point (Figure 2D), were necessary for the activation of infiltrating myeloid cells.

In an attempt to prevent some of the interactions between myeloid cells and T cells, IFNγKO mice were treated with the compound vaccine. In IFNγKO mice, tumor regression was prevented (Figure 4E) and the activation of myeloid cells (i.e., upregulation of MHC II) was markedly decreased compared to that observed in wild type mice (Figure 4F). These results indicate that efficient activation of F4/80^+^ myeloid cells, presumably by CD8^+^ T cells, is dependent on IFNγ.

We further characterized the activation status of myeloid cells infiltrating the tumor. The early increase in *Ly6C* gene expression in the tumor (before day 8) was accompanied with the expression of some myeloid markers upregulated during acute inflammation and commonly associated with M1-polarized macrophages (i.e. MHC II^hi^, TNFα^hi^, CD86^hi^, Figure S4). However, the expression peak for several other markers of inflammatory cells was only reached later: see e.g. the abundance of CCL2 and CCR2^+^ cells (peaking at day 10), or iNOS, a key M1 marker whose expression peaked at day 11, when the tumors had already markedly regressed (Figure S4). We examined additional genes initially associated with M2 cells (Fizz, IL-10, CD206, TGF-β), and observed an early and marked increase for some of them (Fizz, IL-10). These data underline that the local delivery of the vaccine+IFNα activated and reprogrammed the myeloid cell compartment with modulation of various genes that no more fit with the polarized M1/M2 schema as underlined recently [20].

**Figure 5:**
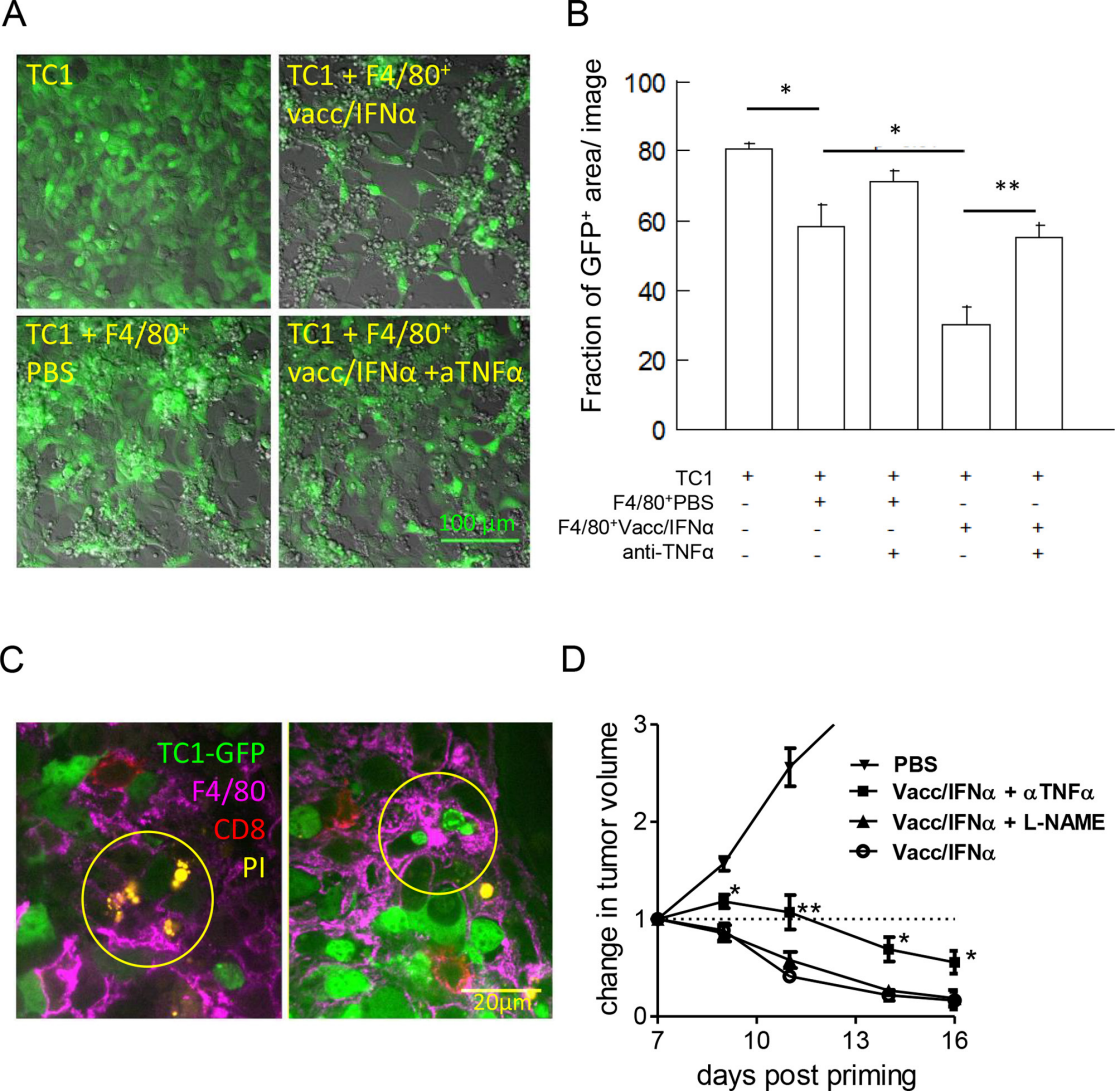
Activated-myeloid cells contribute to the killing of TC1-tumor cells by TNF-α release **A.** TC1-GFP^+^ tumor cells were co-cultured with F4/80^+^ cells isolated from tumors at day 8. Contrary to control tumors (lower left), myeloid cells from vaccine treated mice (right panels) were cytotoxic for tumor cells (disappearance of GFP labeling, loss of tumor cells). Cytotoxic activity of F4/80^+^ cells was blocked in the presence of anti-TNFα Ab (lower right). Representative examples out of 3 independent experiments are shown. **B.** Quantification of the living cells, measured in one out of 3 independent experiments. The apparent fraction of living adherent TC1 cells (fraction of the image occupied by GFP^+^ cells, measured in 6–17 images per condition) is shown in the various conditions. **C.** Example of dying tumor cells in tumors of vaccinated mice 1 day after the boost, where PI^+^ GFP^−^ cells were found in close contact with F4/80^+^ cells (left) or living tumor cells (small round GFP^++^ cells) were found within F4/80^+^ cells (right). **D.** The efficacy of E7-vaccine + IFNα treatment is partially reduced in anti-TNFα treated mice but was not affected by NO inhibition with L-NAME.

To determine how tumor-infiltrating myeloid cells could contribute to tumor regression, we first looked at the production of TNFα by myeloid cells. About 12% of CD11b^+^ cells infiltrating regressing tumors in vaccinated mice spontaneously produced TNFα *ex vivo*, compared to 3% of these cells in tumors from control mice (Figure S5). Stimulation with IFNγ+LPS *in vitro* revealed that the vast majority of CD11b^+^ cells (>80%) from vaccinated mice readily produced TNFα. These results are suggestive of a cytotoxic potential for these myeloid cells infiltrating regressing tumors. In co-culture experiments, TC1-GFP^+^ cells were effectively killed by F4/80^+^ cells purified from regressing tumors, as detected by the reduced density of adherent TC1 cells (Figure 5A and 5B). In sharp contrast, a dense network of healthy TC1-GFP^+^ cells was observed in co-cultures with myeloid cells from progressing tumors. Importantly, we could show that the cytotoxic activity of myeloid cells was partially blocked in the presence of an anti-TNFα antibody (Figure 5A and 5B). Of note, in this *in vitro* assay, CD8 T cells, purified from tumor-bearing-mice that were vaccinated 8 days earlier, did not modify the cytotoxicity of purified macrophages. CD8 T cells were added at densities 10 times lower than that of monocytes/macrophages, which corresponds to *in vivo* ratios (data not shown). The CD8-macrophage cooperation observed *in vivo* presumably required precise spatio-temporal constraints that were not recapitulated in the *in vitro* assay.

Next, we attempted to visualize directly dying tumor cells in the tumor tissue of vaccinated animals: in tumors examined 24 h after the boost, most dead (PI^+^) tumor cells were found surrounded by F4/80^+^ myeloid cells (Figure 5C, left panel), at several cell diameters from CD8^+^ T cells. In addition, we frequently observed examples of living (still green) but compacted TC1 cells fully engulfed by myeloid cells (Figure 5C, right panel). These results revealed that at least a fraction of myeloid cells that infiltrate regressing tumors were likely to be cytotoxic for TC1-tumor cells by mechanisms that could include TNFα release and phagocytosis.

To gain further insight into the anti-tumoral *in vivo* mechanisms, we blocked potential myeloid effector molecules, TNFα and NO, in vaccinated TC1-bearing mice. As suggested by *in vitro* experiments, an anti-TNFα treatment, but not L-NAME treatment (an inhibitor of NO production), partially inhibited vaccination-induced tumor regression (Figure 5D).

Altogether, these results show that a multi-step CD8^+^ T cell / myeloid-cell cooperation takes place within the tumor microenvironment after vaccination : this dynamic cooperation allows that vaccine-elicited CD8 T cells and activated cytotoxic myeloid-cells contribute to tumor regression.

## DISCUSSION

CSF-1 overexpression and high densities of intra-tumoral macrophages are often considered as indicators of poor prognosis for cancer patients [21, 22]. Even if this is true in progressing tumors, this should not hide the fact that appropriately stimulated macrophages may directly or indirectly slow down tumor growth [23, 24]. Here, we have shown that *after an appropriate stimulation*, intra-tumoral macrophages - in cooperation with T cells - contributed efficiently to tumor regression. This apparent discrepancy (pro- or anti-tumoral macrophages) illustrates the importance of taking into account the interactions between different immune cells, and the temporal dimension of an immune response, which allows the apparently same cell type to exert quite different effects [6].

More precisely, we have shown that rejection of TC1 and EG7 solid tumors induced by a vaccine+IFNα requires the participation of activated-myeloid cells. Our study highlights the anti-tumoral potential of macrophages during an acute immune response induced in tumors. Such an anti-tumoral potential has already been reported, but after a targeted macrophage activation [23, 25, 26] and rarely following cooperation with T cells [27–29]. What was the appropriate monocyte/macrophage stimulation in our experiments? Both components of the vaccine (IFNα and STxB-vaccine) probably contributed to it. IFNα is known to be able to trigger an inflammatory response [29, 30] and IFN type I downstream signaling was recently associated with efficient antitumor activity in some chemotherapy-induced immune responses [31]. The contribution of STxB-vaccine to the appropriate myeloid stimulation could have lied in the activation of a CD8^+^ T cell response associated with an increase in IFNγ. IFNγ is a potent stimulus for myeloid cells, especially when combined with CD40 stimulation [32]. Such a coupled IFNγ plus CD40 stimulation may occur during cognate T cell-myeloid cell close contacts. The existence of such a phenomenon is suggested by different observations. 1) Signs of myeloid cell activation (MHC II and CD86 expression, Figure 4C and S4) took place with the first wave of T cell infiltration (5 days after the priming), quantitatively quite modest, but potentially qualitatively important. 2) Intra-tumoral T cell-macrophage interactions were easily observed at day 5, despite the rarity of infiltrating T cells at that time (Figure 2C and 2D). 3) T cells played an important role even when they were not abundant, e.g., in CXCR3 KO mice. 4) Macrophage activation, measured by MHC II expression, did not occur under conditions in which activated-T cells were absent (e.g., after CD8^+^ T cell depletion, Figure 4C). 5) IFNγ played a key role in vaccine efficacy, potentially and at least in part, through T cell-induced macrophage activation (Figure 4E and 4F). This T cell / myeloid cells cooperation is reminiscent of the activation of host macrophages reported after adoptive cell transfer of IFNγ-producing T cells [10, 28, 33, 34, 35]. However, here, it has not been observed as a result of the massive introduction of IFNγ-producing T cells but simply as a result of T-cell stimulation in parallel to mimicking a local infection.

The efficient priming of E7-specific CD8^+^ T cells, which is necessary to achieve tumor regression, was made possible by the use of the STxB-vaccine that favors induction of CD8^+^ T cells [36, 37], in the absence of a marked CD4 activation. In our compound vaccine, it is likely that CD8^+^ T cell help and recruitment were provided by IFNα-dependent responses. There is an apparent paradox in the fact that tumor regression was blocked by complete CD8^+^ T cells depletion with anti-CD8 antibodies, but remained unaffected when tumor-infiltrating CD8 T cells were markedly depleted in CXCR3KO mice. Different and non-exclusive explanations are possible. One is that the consequences of a full depletion are quite distinct from those of a partial depletion, especially if T cells act as a trigger or an amplifier for other cytotoxic effectors, as discussed below. Secondly, tumor regression could be conditioned by CD8^+^ T cell/myeloid cell interactions taking place in tumor-draining lymph nodes, a phenomenon not expected to be affected in CXCR3 KO mice. Finally, CD8^+^ T cells may include two subsets, one of which being independent of CXCR3 ligands, for instance depending on CCL5 for its infiltration.

After treatment with the compound vaccine, the first sign of local anti-tumoral response was an increase in CD11b^+^ myeloid cells, associated with high intra-tumoral levels of *Ly6C* and *CCL2* gene expression, typical of the appearance of classical/inflammatory monocytes recruited by CCL2/CCR2 [38]. This recruitment is functionally important, since we have shown that TC1-tumor regression was blocked in CCR2KO mice. A crucial difference with monocytes infiltrating progressing tumors [39, 40] is the fact that the vaccination promoted an inflammatory microenvironment. In our settings, IFNα induces the production of CXCL10. The fact that the depletion of myeloid cells with PLX3397 decreases the T cell infiltrate strongly suggests that myeloid cells act upstream to attract T cells to the tumor. In return, the coexistence of a small but decisive amount of T cells at the time of myeloid cell infiltration might have driven the swift up regulation of MHC class II, CD86 and TNFα in myeloid cells. The emergence of such activated myeloid cells is reminiscent of the TipDC observed after bacterial infection [41] or the CD11b^+^CD11c^+^Ly6C^hi^ cells infiltrating tumors treated with anthracycline [42]. Note that the coexpression of F4/80 and CD11c in a fraction of activated intra-tumoral myeloid cells obscures the distinction between macrophages and dendritic cells in this microenvironment (contrary to the situation observed in lymph nodes). In order to mimic the local activation of macrophages by infiltrating T cells, we performed intra-tumoral injections of an anti-CD40 agonist antibody + IFNγ. However, this treatment elicited regression / stabilization in only a minority (4 out of 12) of TC1 tumors (data not shown). Thus, this combined treatment did not faithfully mimic the spatio-temporal features of the synchronized recruitment of T cells and monocytes elicited by the vaccine compound.

Myeloid cells could mediate cytotoxic and/or cytostatic effects on malignant cells. Our *in vitro* and *in vivo* data show that F4/80^+^ cells from vaccinated mice can directly kill TC1 tumor cells by mechanisms that involve phagocytosis and TNFα. In previous studies with anti-tumoral macrophages, NO, ROS or TNFα were shown to slow down tumor growth *in vitro*, which may reveal cytostatic rather than cytotoxic effects [10, 26, 43, 44]. Even if iNOS^+^ macrophages could be necessary in other settings for an optimal recruitment of T cells into tumors [24], in our system of vaccination against TC1 tumors, NO production was dispensable for vaccine-induced tumor regression.

When macrophages are considered exclusively as pro-tumoral cells, it is logical to aim at depleting them so as to facilitate the efficacy of cytotoxic agents or cytotoxic T cells [18, 45]. It has been reported that macrophage stimulation with poly I:C triggers an antitumor cytotoxicity [23], and that local low-dose gamma irradiation activated TAM and resulted in the recruitment of cytotoxic T cells [24]. We propose a more global view, in which myeloid cells, when appropriately stimulated, can be activated by primed T cells and can support those T cells to directly eliminate tumor cells. The existence of a bi-directional interaction between myeloid cells and T cells in the tumor, including attraction and activation, warrants further investigations.

Our findings contrast with recent reports in which myeloid cells reduce the anti-tumoral action of chemotherapy or adoptive T cell transfer [18, 45, 46]. This paradox most probably reflects the heterogeneity of the tumor types and microenvironments and the need to identify which therapies or treatment-schedule conditions the generation of anti-tumor myeloid cells.

As mentioned earlier, a few studies report on the existence of anti-tumoral macrophages. However, to our knowledge, this is the first report in which an efficient anti-infectious response is used as a conceptual model for an anti-tumoral response. In a typical anti-infectious response the local alert of resident macrophages results in macrophage activation and recruitment of more innate cells followed by T cell priming and antigen-specific T cell recruitment to the infection site. By making the appropriate spatio-temporal measurements, we were able to describe an anti-tumoral response with the initial local activation of myeloid cells, involving a small amount of tumor-infiltrating T cells, which clearly preceded a more massive T cell infiltrate immediately after the vaccination boost. Our study highlights that the anti-tumoral myeloid cells should not be viewed as a static subset entirely distinct from the pro-tumoral ones, but rather as a dynamic population resulting from an influx of myeloid cells and from their intra-tumoral differentiation. The kinetics of these fluxes would deserve a whole additional study.

## MATERIALS AND METHODS

### Animal studies

C57BL/6J mice were purchased from Charles River laboratories. Mice were maintained in the Cochin Institute SPF animal facility. Animal care was performed by expert technicians in compliance with the Federation of European Laboratory Animal science association and under the approval of the animal experimentation ethics committee of Paris Descartes (CEEA34.ED.042.12).

The TC1 cell line, and for some experiments the EG7 cell line, were maintained in culture in complete RPMI, including 10% FCS (GE Healthcare), antibiotics (Penicillin 50U/ml, Streptomycin 50 μg/ml, GIBCO), L-Glutamine (4 mM, GIBCO) and Sodium Pyruvate (1 mM, GIBCO). The TC1-GFP cell line was generated by infection of TC1 cells with a GFP-lentivirus. Lentiviral production was obtained by transient calcium-phosphate co-transfection of the 293T cell line with the TRIPΔU3-EF1α vector, the p8.91 encapsidation vector (ΔVprΔVifΔVpuΔNef), and a vesicular stomatitis virus-G protein (VSV-G) envelop expression plasmid (pHCMV-G). GFP was expressed by >90% of TC1 cells.

Eight weeks-old C57BL/6J were inoculated in the flank with 10^5^ TC1 cells. When tumors reached a diameter of 6 mm (∼10 days), mice were primed (= day 0) with a peritumoral injection of 20 μg of E7-vaccine (STxBE7 vaccine, [16]) and 6 × 10^5^U of IFNα_4_ [46] in a total volume of 200 μl. Littermates were injected with PBS as control. The next day, vaccinated mice received the same dose of IFNα. This protocol was repeated one week later (boost, = day7). For some experiments, IFNγKO (kindly provided by O. Lantz, Institut Curie, Paris, France), CXCR3KO mice (SigN, Singapore) and CCR2KO mice (kindly provided by T. Henry, CIRI Inserm, Lyon, France) were used.

For CD8^+^ T cell depletion, 200 μg of anti-CD8 antibody (BioXcell, clone 53–6.72) were injected every 2 days starting on day 5 after the priming. Anti-CD4 antibody (BioXcell, clone GK1.5, 150 μg) or anti-TNFα (BioXcell, clone TN3–19.12, 300 μg), were injected every 3 days starting at the time of priming. Macrophage depletion was performed with PLX3397 (Plexxikon), a CSF1-R signaling inhibitor. Mice were fed with chow containing PLX3397, or control chow, right after the priming. With such a treatment, macrophage depletion is expected to begin 2–3 days later [18]. Nitric oxide (NO) was blocked with L-NAME added in the drinking water (500 μg/ml, N5751 Sigma,) the day of priming and renewed every 2 days.

### Analysis of the tumor infiltrate by multicolor flow cytometry

Fresh TC1 tumors were dissociated mechanically and incubated 30 minutes at 37°C with DNaseI (100 μg/ml, Roche) and collagenase (1 mg/ml, Roche). The cell suspension was filtered on a 40 μm sieve, and rinsed several times with PBS 2% FCS 0.5 mM EDTA. Cells (4 × 10^6^) were stained in 96-wells round bottom plates with dead/live staining (Blue fluorescent reactive dye, Invitrogen) during 20 minutes at room temperature. Fc receptors were blocked with anti-FCR (anti-CD16 CD-32 at 5 μg/ml, BD Pharmingen). Then, cells were either stained with biotinylated anti-Ly6C (myeloid analysis) or biotinylated anti-TCRβ (lymphoid analysis), for 15 minutes at 4°C with agitation. After 2 washes in PBS 2% FCS, cells were stained with antibodies CD45.2-PerCP-Cy5.5, CD11b-APC, CD11c-PE-Cy7, NK1.1-PE, streptavidin-Pac Blue and Ly6G-FITC (for myeloid staining), antibodies CD45.2-APC, IA/IE-PE, CD11b-FITC, CD11c-PE-Cy7, streptavidin-Pacific Blue and F4/80-PerCP-Cy5.5 (for macrophages and MHC II staining), or with antibodies CD45.2-APC, CD4-Pacific Blue, CD8-APC-H7, streptavidin-PE-Cy7, CD19-FITC, NK1.1-PerCP-Cy5.5 and anti-TCRβ-PE (for lymphoid staining). Antibodies were purchased from BD Pharmingen, except for the CD11b-APC antibody, which was purchased from eBiosciences. For detection of E7-specific CD8^+^ T cells, cell suspensions were stained with PE-Kb/E7 dextramer (JA2195, Immudex) for 40 minutes at 4°C, followed by staining with cell surface markers for CD8, CD4, NK, CD19 and CD45. After washing in PBS, cells were fixed in 1% PFA, stored at 4°C, and acquired the next day with LSR II flow cytometer (BD Bioscience).

### Immunofluorescence

Tumor pieces were fixed overnight with Periodate-Lysine-Paraformaldehyde [48] at 4°C and Immunofluorescence on tumor slices was performed as previously described [17, 49]. Immunostaining was performed by first blocking Fc receptors with anti-FCR (BD Pharmingen), then staining for 1 h RT or at 4°C overnight was performed with primary antibodies specific for CD8-PerCP, CD45-APC, IA-IE-PE, CD11b-FITC, Ly6C-APC, CD31-biotin (all from BD Pharmingen), fibronectin, gp38/podoplanin, F4/80-biotin (all from Biolegend) or F4/80-PE (AbD Serotec). Immunodetection was performed using anti-Rat or anti-rabbit antibodies coupled to 488, 568 or 647 (BD Pharmingen) and streptavidin- Alexa Fluor 488 or 647 (Invitrogen). Slices were then counter-stained with Hoechst for 10 min at room temperature. Antibodies were diluted in PBS, 0.5% BSA, 2% human serum. Images were obtained with a spinning disk microscope equipped with a CoolSnap HQ2 camera (Photometrics) and a 20x and a 63x objective. All images were acquired with MetaMorph 7 imaging software (Molecular Devices) and analysed with ImageJ. For staining of dying cells *in vivo*, vaccinated TC1-GFP bearing mice received an intra-tumoral injection of propidium iodide (1 mg/ml) and were sacrificed 30 min later.

### Transcriptomic analysis

Tumor RNA was extracted using RNeasy Mini Kit (Qiagen) according to the manufacturer's instructions and gene expression was analyzed with the nanostring technology. 36 lanes of Nanostring data were processed using Accelrys Pipeline Pilot. The geometrical mean of the positive control probe counts were computed for each lane and a scaling factor computed for each lane being the average of the geometrical means of all lanes A was divided by the geometrical mean of that particular lane. This scaling factor was then applied to all probe counts for all lanes as a means to normalize for the technical variability of the platform. The house keeping genes *ACTB, GAPDH* and *RPL4* were then used to normalize for any RNA loading differences. This was performed in the same manner as the positive control probes where the scaling factor was computed from the geometrical mean of the housekeeping genes. The positive control and housekeeping normalized counts were then logarithmically transformed and used for all subsequent analysis. One way ANOVAs were used to test if any of the probes counts were significantly different between sample groups. Pearson correlations were to identify pairs of correlating genes. All statistical testing was done using the R statistical language version 2.15.2. *p* < 0.05 were deemed to be statistically significant. Multiple testing corrections were performed for all tests using the method of Benjamini and Hochberg. Visualization of the data and test results were done using GraphPad Prism 5.

### Cytotoxic assay with tumor-derived myeloid cells

F4/80^+^ myeloid cells were purified from tumor cell suspensions using F4/80-biotin Ab (eBioscience), and streptavidin-beads before sorting with Automacs (Miltenyi Biotec). Purified cells were treated 5 minutes at 37°C with DNase (100 μg/ml) to remove cell debris. F4/80^+^ cell suspension were > 90% pure. Purified myeloid cells (2 × 10^5^) were added on the top of a monolayer of TC1-GFP^+^ cells (2 × 10^4^ cells) and co-cultured for 48 h at 37°C. TNFα was blocked with anti-TNFα at 10 μg/ml (Abcam). Images were obtained with an inverted microscope (TE2000-E; Nikon) equipped with a 20x objective, and Metamorph imaging software.

### Statistics

Unless otherwise indicated, results are expressed as means ± SEM of 3 to 6 mice. All experiments were repeated at least twice, yielding similar results. Data were analyzed with GraphPad Prism5 software. *p* values were calculated by the unpaired Student's t test or One-way ANOVA and Tukey test for multiple comparison. Values ≤ 0.05 were considered significant. *p < 0.05; **p < 0.01; ***p < 0.001.

## SUPPLEMENTARY MATERIAL FIGURES



## Abbreviations

TAM: tumor-associated macrophages
TIL: tumor-infiltrating T lymphocytes

## References

[1] Galon J, Costes A, Sanchez-Cabo F, Kirilovsky A, Mlecnik B, Lagorce-Pagès C, Tosolini M, Camus M, Berger A, Wind P, Zinzindohoué F, Bruneval P, Cugnenc P-H (2006). Type, Density, and Location of Immune Cells Within Human Colorectal Tumors Predict Clinical Outcome. Science.

[2] Eyles J, Puaux A-L, Wang X, Toh B, Prakash C, Hong M, Tan TG, Zheng L, Ong LC, Jin Y, Kato M, Prévost-Blondel A, Chow P (2010). Tumor cells disseminate early, but immunosurveillance limits metastatic outgrowth, in a mouse model of melanoma. J Clin Invest.

[3] Rosenberg SA, Yang JC, Sherry RM, Kammula US, Hughes MS, Phan GQ, Citrin DE, Restifo NP, Robbins PF, Wunderlich JR, Morton KE, Laurencot CM, Steinberg SM (2011). Durable Complete Responses in Heavily Pretreated Patients with Metastatic Melanoma Using T-Cell Transfer Immunotherapy. Clin Cancer Res.

[4] Grupp SA, Kalos M, Barrett D, Aplenc R, Porter DL, Rheingold SR, Teachey DT, Chew A, Hauck B, Wright JF, Milone MC, Levine BL, June CH (2013). Chimeric Antigen Receptor-Modified T Cells for Acute Lymphoid Leukemia. N Engl J Med.

[5] Wolchok JD, Kluger H, Callahan MK, Postow MA, Rizvi NA, Lesokhin AM, Segal NH, Ariyan CE, Gordon R-A, Reed K, Burke MM, Caldwell A, Kronenberg SA (2013). Nivolumab plus Ipilimumab in Advanced Melanoma. N Engl J Med.

[6] Bercovici N, Trautmann A (2012). Revisiting the role of T cells in tumor regression. Oncoimmunology.

[7] Hoption Cann SA, van Netten JP, van Netten C (2003). Dr William Coley and tumour regression: a place in history or in the future. Postgrad Med J.

[8] Boissonnas A, Fetler L, Zeelenberg IS, Hugues S, Amigorena S (2007). *In vivo* imaging of cytotoxic T cell infiltration and elimination of a solid tumor. J Exp Med.

[9] Breart B, Lemaitre F, Celli S, Bousso P (2008). Two-photon imaging of intratumoral CD8+ T cell cytotoxic activity during adoptive T cell therapy in mice. J Clin Invest.

[10] Hollenbaugh JA, Reome J, Dobrzanski M, Dutton RW (2004). The rate of the CD8-dependent initial reduction in tumor volume is not limited by contact-dependent perforin, Fas ligand, or TNF-mediated cytolysis. J Immunol.

[11] Prevost-Blondel A, Neuenhahn M, Rawiel M, Pircher H (2000). Differential requirement of perforin and IFN-gamma in CD8 T cell-mediated immune responses against B16.F10 melanoma cells expressing a viral antigen. Eur J Immunol.

[12] Salmon H, Franciszkiewicz K, Damotte D, Dieu-Nosjean MC, Validire P, Trautmann A, Mami-Chouaib F, Donnadieu E (2012). Matrix architecture defines the preferential localization and migration of T cells into the stroma of human lung tumors. J Clin Invest.

[13] Mrass P, Takano H, Ng LG, Daxini S, Lasaro MO, Iparraguirre A, Cavanagh LL, Andrian UH von, Ertl HCJ, Haydon PG, Weninger W (2006). Random migration precedes stable target cell interactions of tumor-infiltrating T cells. J Exp Med.

[14] Boissonnas A, Licata F, Poupel L, Jacquelin S, Fetler L, Krumeich S, Théry C, Amigorena S, Combadière C (2013). CD8+ Tumor-Infiltrating T Cells Are Trapped in the Tumor-Dendritic Cell Network. Neoplasia NYN.

[15] Engelhardt JJ, Boldajipour B, Beemiller P, Pandurangi P, Sorensen C, Werb Z, Egeblad M, Krummel MF (2012). Marginating Dendritic Cells of the Tumor Microenvironment Cross-Present Tumor Antigens and Stably Engage Tumor-Specific T Cells. Cancer Cell.

[16] Vingert B, Adotevi O, Patin D, Jung S, Shrikant P, Freyburger L, Eppolito C, Sapoznikov A, Amessou M, Quintin-Colonna F, Fridman WH, Johannes L, Tartour E (2006). The Shiga toxin B-subunit targets antigen *in vivo* to dendritic cells and elicits anti-tumor immunity. Eur J Immunol.

[17] Asperti-Boursin F, Real E, Bismuth G, Trautmann A, Donnadieu E (2007). CCR7 ligands control basal T cell motility within lymph node slices in a phosphoinositide 3-kinase- independent manner. J Exp Med.

[18] DeNardo DG, Brennan DJ, Rexhepaj E, Ruffell B, Shiao SL, Madden SF, Gallagher WM, Wadhwani N, Keil SD, Junaid SA, Rugo HS, Hwang ES, Jirström K (2011). Leukocyte complexity predicts breast cancer survival and functionally regulates response to chemotherapy. Cancer Discov.

[19] Yang X, Lu P, Ishida Y, Kuziel WA, Fujii C, Mukaida N (2006). Attenuated liver tumor formation in the absence of CCR2 with a concomitant reduction in the accumulation of hepatic stellate cells, macrophages and neovascularization. Int J Cancer J Int Cancer.

[20] Xue J, Schmidt SV, Sander J, Draffehn A, Krebs W, Quester I, De Nardo D, Gohel TD, Emde M, Schmidleithner L, Ganesan H, Nino-Castro A, Mallmann MR (2014). Transcriptome-based network analysis reveals a spectrum model of human macrophage activation. Immunity.

[21] Lin EY, Nguyen AV, Russell RG, Pollard JW (2001). Colony-Stimulating Factor 1 Promotes Progression of Mammary Tumors to Malignancy. J Exp Med.

[22] Pollard JW (2009). Trophic macrophages in development and disease. Nat Rev Immunol.

[23] Shime H, Matsumoto M, Oshiumi H, Tanaka S, Nakane A, Iwakura Y, Tahara H, Inoue N, Seya T (2012). Toll-like receptor 3 signaling converts tumor-supporting myeloid cells to tumoricidal effectors. Proc Natl Acad Sci.

[24] Klug F, Prakash H, Huber PE, Seibel T, Bender N, Halama N, Pfirschke C, Voss RH, Timke C, Umansky L, Klapproth K, Schäkel K, Garbi N (2013). Low-dose irradiation programs macrophage differentiation to an iNOS+/M1 phenotype that orchestrates effective T cell immunotherapy. Cancer Cell.

[25] Beatty GL, Chiorean EG, Fishman MP, Saboury B, Teitelbaum UR, Sun W, Huhn RD, Song W, Li D, Sharp LL, Torigian DA, O’Dwyer PJ, Vonderheide RH (2011). CD40 Agonists Alter Tumor Stroma and Show Efficacy Against Pancreatic Carcinoma in Mice and Humans. Science.

[26] Buhtoiarov IN, Sondel PM, Wigginton JM, Buhtoiarova TN, Yanke EM, Mahvi DA, Rakhmilevich AL (2011). Anti-tumour synergy of cytotoxic chemotherapy and anti-CD40 plus CpG-ODN immunotherapy through repolarization of tumour-associated macrophages: Chemotherapy and immunotherapy synergize in Mϕ activation. Immunology.

[27] Haabeth OAW, Lorvik KB, Hammarström C, Donaldson IM, Haraldsen G, Bogen B, Corthay A (2011). Inflammation driven by tumour-specific Th1 cells protects against B-cell cancer. Nat Commun.

[28] Vicetti Miguel RD, Cherpes TL, Watson LJ, McKenna KC (2010). CTL induction of tumoricidal nitric oxide production by intratumoral macrophages is critical for tumor elimination. J Immunol Baltim Md 1950.

[29] Chmielewski M, Kopecky C, Hombach AA, Abken H (2011). IL-12 Release by Engineered T Cells Expressing Chimeric Antigen Receptors Can Effectively Muster an Antigen-Independent Macrophage Response on Tumor Cells That Have Shut Down Tumor Antigen Expression. Cancer Res.

[30] Bogdan C, Mattner J, Schleicher U (2004). The role of type I interferons in non-viral infections. Immunol Rev.

[31] Hervas-Stubbs S, Perez-Gracia JL, Rouzaut A, Sanmamed MF, Le Bon A, Melero I (2011). Direct effects of type I interferons on cells of the immune system. Clin Cancer Res Off J Am Assoc Cancer Res.

[32] Sistigu A, Yamazaki T, Vacchelli E, Chaba K, Enot DP, Adam J, Vitale I, Goubar A, Baracco EE, Remédios C, Fend L, Hannani D, Aymeric L (2014). Cancer cell-autonomous contribution of type I interferon signaling to the efficacy of chemotherapy. Nat Med.

[33] Heusinkveld M, van der Burg SH (2011). Identification and manipulation of tumor associated macrophages in human cancers. J Transl Med.

[34] Hollenbaugh JA, Dutton RW (2006). IFN-γ Regulates Donor CD8 T Cell Expansion, Migration, and Leads to Apoptosis of Cells of a Solid Tumor. J Immunol.

[35] Textor A, Listopad JJ, Wührmann LL, Perez C, Kruschinski A, Chmielewski M, Abken H, Blankenstein T, Charo J (2014). Efficacy of CAR T-cell therapy in large tumors relies upon stromal targeting by IFNγ. Cancer Res.

[36] Adotevi O, Vingert B, Freyburger L, Shrikant P, Lone Y-C, Quintin-Colonna F, Haicheur N, Amessou M, Herbelin A, Langlade-Demoyen P, Fridman WH, Lemonnier F, Johannes L (2007). B subunit of Shiga toxin-based vaccines synergize with alpha-galactosylceramide to break tolerance against self antigen and elicit antiviral immunity. J Immunol Baltim Md 1950.

[37] Sandoval F, Terme M, Nizard M, Badoual C, Bureau M-F, Freyburger L, Clement O, Marcheteau E, Gey A, Fraisse G, Bouguin C, Merillon N, Dransart E (2013). Mucosal Imprinting of Vaccine-Induced CD8+ T Cells Is Crucial to Inhibit the Growth of Mucosal Tumors. Sci Transl Med.

[38] Lee H-W, Choi H-J, Ha S-J, Lee K-T, Kwon Y-G (2013). Recruitment of monocytes/macrophages in different tumor microenvironments. Biochim Biophys Acta BBA - Rev Cancer.

[39] Egeblad M, Ewald AJ, Askautrud HA, Truitt ML, Welm BE, Bainbridge E, Peeters G, Krummel MF, Werb Z (2008). Visualizing stromal cell dynamics in different tumor microenvironments by spinning disk confocal microscopy. Dis Model Mech.

[40] Qian B-Z, Li J, Zhang H, Kitamura T, Zhang J, Campion LR, Kaiser EA, Snyder LA, Pollard JW (2011). CCL2 recruits inflammatory monocytes to facilitate breast-tumour metastasis. Nature.

[41] Serbina NV, Salazar-Mather TP, Biron CA, Kuziel WA, Pamer EG (2003). TNF/iNOS-Producing Dendritic Cells Mediate Innate Immune Defense against Bacterial Infection. Immunity.

[42] Ma Y, Mattarollo SR, Adjemian S, Yang H, Aymeric L, Hannani D, Catani JPP, Duret H, Teng MWL, Kepp O, Wang Y, Sistigu A, Schultze JL (2014). CCL2/CCR2-Dependent Recruitment of Functional Antigen-Presenting Cells into Tumors upon Chemotherapy. Cancer Res.

[43] Lum HD, Buhtoiarov IN, Schmidt BE, Berke G, Paulnock DM, Sondel PM, Rakhmilevich AL (2006). Tumoristatic effects of anti-CD40 mAb-activated macrophages involve nitric oxide and tumour necrosis factor-alpha. Immunology.

[44] Pommier A, Audemard A, Durand A, Lengagne R, Delpoux A, Martin B, Douguet L, Campion AL, Kato M, Avril M-F, Auffray C, Lucas B, Prévost-Blondel A (2013). Inflammatory monocytes are potent antitumor effectors controlled by regulatory CD4+ T cells. Proc Natl Acad Sci.

[45] Mok S, Koya RC, Tsui C, Xu J, Robert L, Wu L, Graeber TG, West BL, Bollag G, Ribas A (2014). Inhibition of CSF-1 Receptor Improves the Antitumor Efficacy of Adoptive Cell Transfer Immunotherapy. Cancer Res.

[46] Sluijter M, van der Sluis TC, van der Velden PA, Versluis M, West BL, van der Burg SH, van Hall T (2014). Inhibition of CSF-1R supports T-cell mediated melanoma therapy. PloS One.

[47] Le Bon A, Etchart N, Rossmann C, Ashton M, Hou S, Gewert D, Borrow P, Tough DF (2003). Cross-priming of CD8+ T cells stimulated by virus-induced type I interferon. Nat Immunol.

[48] Kastenmüller W, Brandes M, Wang Z, Herz J, Egen JG, Germain RN (2013). Peripheral Prepositioning and Local CXCL9 Chemokine-Mediated Guidance Orchestrate Rapid Memory CD8+ T Cell Responses in the Lymph Node. Immunity.

[49] Wang SF, Fouquet S, Chapon M, Salmon H, Regnier F, Labroquere K, Badoual C, Damotte D, Validire P, Maubec E, Delongchamps NB, Cazes A, Gibault L (2011). Early T Cell Signalling Is Reversibly Altered in PD-1 T Lymphocytes Infiltrating Human Tumors. PLoS One.

