# Supplementary material for: Vaccine-induced tumor regression requires a dynamic cooperation between T cells and myeloid cells at the tumor site
Source: Oncotarget. 2015 Aug 11;6(29):27832–46. doi: 10.18632/oncotarget.4940 (PMC4695029; doi:10.18632/oncotarget.4940)
Supplement: Supplementary file 1 [file oncotarget-06-27832-s001.pdf]

SUPPLEMENTARY FIGURES

A

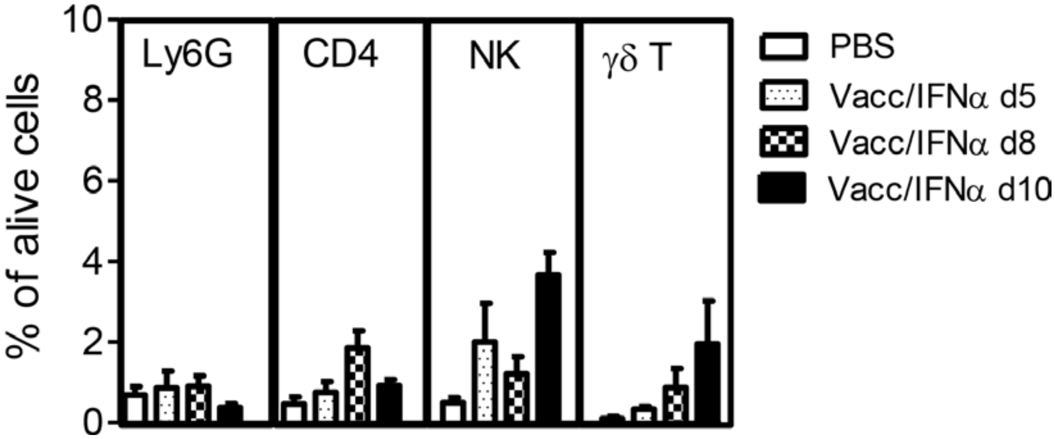

Supplementary Figure S1: Several immune cell subsets (Ly6G⁺ cells, CD4⁺ T cells, NK cells, and γδT cells) remained at low levels during the course of the treatment.

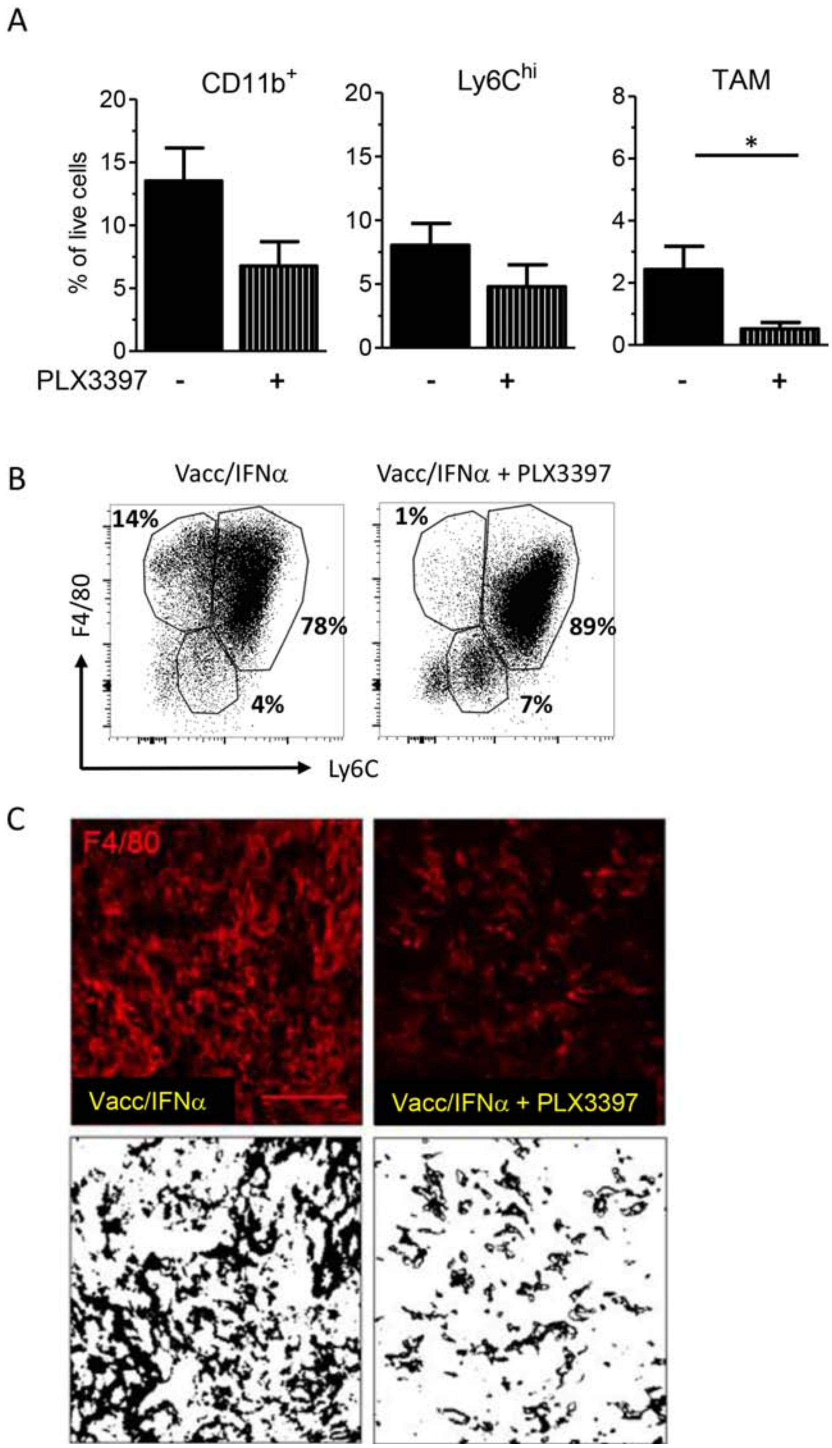

Supplementary Figure S2: A. % of myeloid cells (CD11b^{+}), and two of its subpopulation, tumor-associated macrophages (TAM F4/80^{hi} Ly6C^{neg}) and inflammatory monocytes (Ly6C^{hi} F4/80^{lo}) out of living cells dissociated from tumors at day 8 and 10 after E7-vaccine+IFNα in mice treated or not with PLX3397. Histograms represent the mean ± SEM of 6–7 mice, from 3 independent experiments. PLX3397 treatment reduced significantly the percentage of TAM (~5-fold) (Unpaired t test with Welch's correction). **B.** Typical examples of myeloid subpopulations highlighting the effect of PLX3397 on TAM in vaccine-treated mice. **C.** In PLX3397-treated mice, 8 days after priming with vacc/IFNα, myeloid cells express lower levels of F4/80 and are less abundant than in Mock, vaccine-treated mice. Bar 50 µm. According to the relative surface covered by F4/80 labeling, as estimated in the lower panels, this abundance is ~6-fold lower after PLX3397 in this experiment.

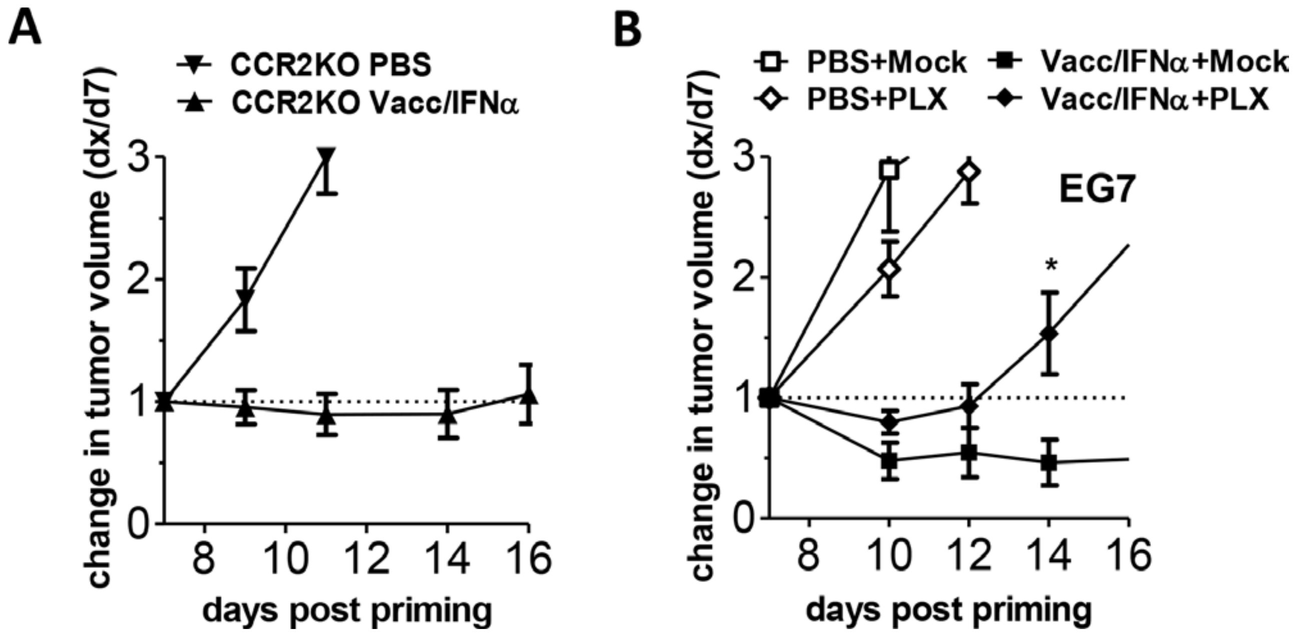

Supplementary Figure S3: Different strategies of myeloid depletion and other tumor models. Tumor evolution is expressed relative to the size at the time of the boost. A. In CCR2KO mice, vaccination induces a stabilization of TC1 tumors, but no longer their regression. **B.** The vaccine-induced regression of EG7 tumors is blocked in PLX-treated mice.

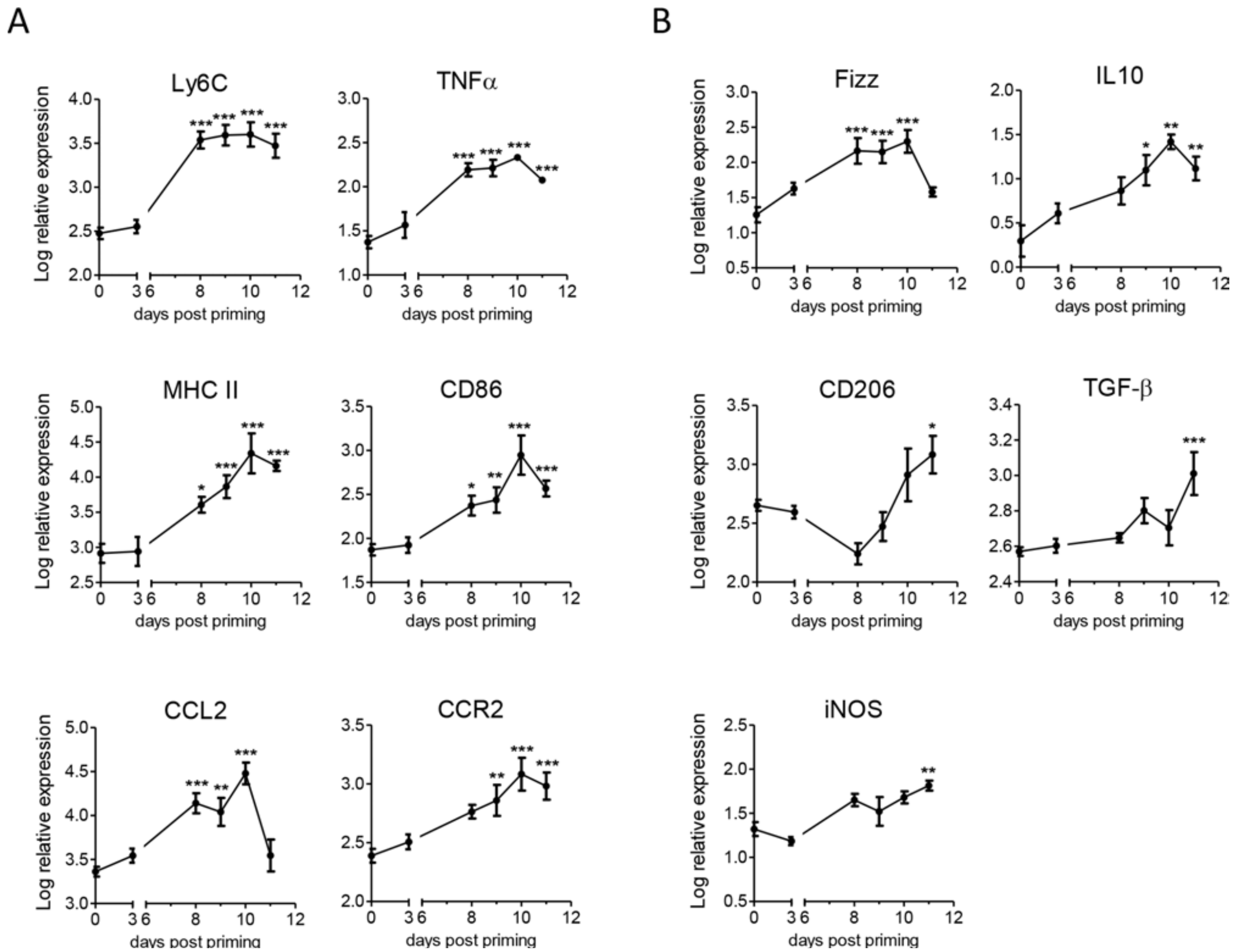

Supplementary Figure S4: E7-vaccine+IFN-α induced an increase in activated myeloid cell infiltrate. A. Transcriptomic analysis showing the upregulation of markers associated with activated monocytes/macrophages in tumors after vaccination. **B.** Progressive upregulation of genes associated with M2 (Fizz, IL-10, CD206, TGF-β) and M1 (iNOS) macrophages during the course of tumor regression.

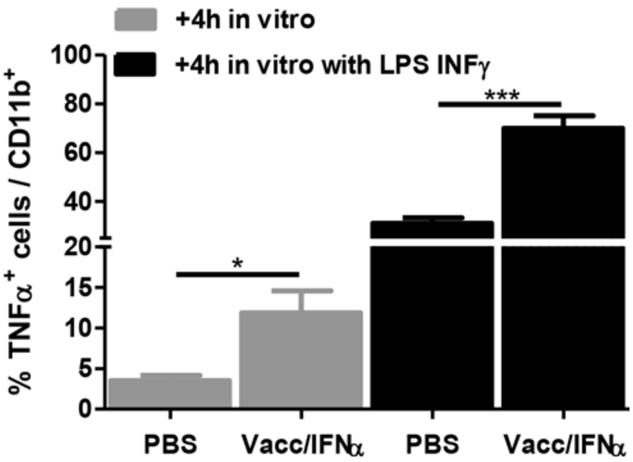

Supplementary Figure S5: Myeloid cells from vaccine-treated mice produced more TNF-α than the PBS control group, both with and without *in vitro* stimulation by LPS + IFNγ. Means of two independent experiments, with 8–10 mice per group.
